# Sex Matters: Robust Sex Differences in Signal Detection in the HIV-1 Transgenic Rat

**DOI:** 10.3389/fnbeh.2017.00212

**Published:** 2017-11-06

**Authors:** Kristen A. McLaurin, Rosemarie M. Booze, Charles F. Mactutus, Amanda J. Fairchild

**Affiliations:** ^1^Program in Behavioral Neuroscience, Department of Psychology, University of South Carolina, Columbia, SC, United States; ^2^Department of Psychology, University of South Carolina, Columbia, SC, United States

**Keywords:** HIV-1 transgenic rat, biological sex, sustained attention, neuroinflammation

## Abstract

Sex differences in human immunodeficiency virus type-1 (HIV-1) have been repeatedly suggested. Females, who account for 51% of HIV-1 seropositive individuals, are inadequately represented in clinical and preclinical studies, as well as in the description of HIV-1 associated neurocognitive disorders (HAND). Direct comparisons of neurocognitive decline in women and men must be made to address this underrepresentation. The effect of biological sex (i.e., the biological factors, including chromosomes and hormones, determining male or female characteristics; WHO, 2017) on sustained attention, which is commonly impaired in HIV-1 seropositive individuals, was investigated in intact HIV-1 transgenic (Tg) and control animals using a signal detection operant task. Analyses revealed a robust sex difference in the rate of task acquisition, collapsed across genotype, with female animals meeting criteria in shaping (at least 60 reinforcers for three consecutive or five non-consecutive sessions) and signal detection (70% accuracy for five consecutive or seven non-consecutive sessions) significantly more slowly than male animals. Presence of the HIV-1 transgene also had a significant effect on shaping and signal detection acquisition, with HIV-1 Tg animals displaying significant deficits in the rate of acquisition relative to control animals–deficits that were more prominent in female HIV-1 Tg animals. Once the animals’ reached asymptotic performance in the signal detection task, female animals achieved a lower percent accuracy across test sessions and exhibited a decreased response rate relative to male animals, although there was no compelling evidence for any effect of transgene. Results indicate that the factor of biological sex may be a moderator of the influence of the HIV-1 transgene on signal detection. Understanding the impact of biological sex on neurocognitive deficits in HIV-1 is crucial for the development of sex-based therapeutics and cure strategies.

## Introduction

Sex differences in human immunodeficiency virus type-1 (HIV-1) have been reported in studies conducted both before (e.g., Melnick et al., 1994) and after the advent of combination antiretroviral therapy (cART; e.g., Hestad et al., 2012; Griesbeck et al., 2016; Royal et al., 2016). Worldwide, women account for approximately 51% of HIV-1 seropositive individuals (women: 17.8 million; men: 17.1 million; UNAIDS, 2016). Young women (ages 15–24) living in sub-Saharan Africa are more than twice as likely as men to be infected with HIV-1 (WHO, 2014). Although women represent a larger population of individuals living with HIV-1, they are inadequately represented in the reports of both clinical and preclinical studies, as well as in the description of HIV-1 associated neurocognitive disorders (HAND; Maki and Martin-Thormeyer, 2009), characterized by deficits in executive function, attention and memory (Cysique et al., 2004; Heaton et al., 2011; Sacktor and Robertson, 2014). Clearly, direct comparisons of neurocognitive decline in women and men must be made to address this underrepresentation (Maki et al., 2015).

Clinical studies directly comparing neurocognitive deficits in HIV-1 seropositive men and women have, thus far, been inconsistent. Early clinical reports observed no sex differences in the progression of neurocognitive impairment (Robertson et al., 2004). In sharp contrast, a pilot study in Zambia, Africa revealed that HIV-1 seropositive women exhibited greater neurocognitive deficits in comparison to HIV-1 seropositive men (Hestad et al., 2012). However, sex-specific differences observed by Hestad et al. (2012) were the result of an unplanned subanalysis, and thus have not been widely acknowledged. More recent clinical studies, conducted in Nigeria, more definitively revealed differential neurocognitive impairment for HIV-1 seropositive women and men (Royal et al., 2016). Specifically, HIV-1 seropositive women, relative to HIV-1 seropositive men, were significantly more impaired on measures of speed of information processing, verbal fluency, learning and memory; deficits which were correlated with higher levels of monocytes (Royal et al., 2016).

Preclinical studies have also provided an opportunity to directly examine the role of biological sex on HAND and its associated neural impairments (Hahn et al., 2015; McLaurin et al., 2016, 2017a; Rowson et al., 2016). In the HIV-1 transgenic (Tg) rat, effects of the HIV-1 transgene were more profound in female animals, relative to male animals, on measures of temporal processing (McLaurin et al., 2016, 2017a), locomotor activity (Rowson et al., 2016), and object recognition memory (Rowson et al., 2016). In sharp contrast, female Tat transgenic mice showed increases in forelimb grip strength and decreases in anxiety-like behavior, assessed using a standard light/dark box test, relative to male Tat transgenic mice (Hahn et al., 2015). Female Tat transgenic mice also displayed significantly lower levels of astrogliosis and significantly more dendritic spines relative to male Tat transgenic mice (Hahn et al., 2015). Differences in the system chosen to model neurocognitive impairment in HIV-1 (i.e., Tat transgenic mice vs. HIV-1 Tg rat) may account for inconsistencies in the observation of sex differences. The present report investigated the effect of biological sex (i.e., the biological factors, including chromosomes and hormones, determining male or female characteristics; WHO, 2017) on sustained attention in the HIV-1 Tg rat, which has been promoted for investigating neurocognitive impairments in HIV-1 in the cART era (review, Vigorito et al., 2015).

Sustained attention, a fundamental component of cognitive capacity, requires individuals to detect rare, unpredictable and weak stimuli over long periods of time (Sarter et al., 2001). Experimental approaches have been developed and validated to assess sustained attention in humans (e.g., Rosvold et al., 1956; Mar et al., 1996) and animals (e.g., McGaughy and Sarter, 1995; Robbins, 1998). Assessments of sustained attention in the present study employed an operant task developed by McGaughy and Sarter (1995). The sustained attention task requires animals to attend to a randomly presented stimulus (i.e., central panel illumination), the presence or absence of which indicates which response to make (i.e., which lever to press) to receive a reinforcer (i.e., sucrose pellet). On each trial, an animal may emit one of four response choices (i.e., hits, misses, correct rejections and false alarms), which indicate the ability to attend to the stimulus (hits, correct rejections), a lapse of attention (misses) or a failure of response inhibition (false alarms). When we began this study, the sustained attention operant task had not been conducted in male and female rats in the same study, allowing for a direct comparison of the factor of biological sex on sustained attention.

Deficits in sustained attention have been previously reported in HIV-1 seropositive children (Watkins et al., 2000) and adults (Fein et al., 1995). In HIV-1 seropositive children, false alarm rates increased throughout the task, suggesting a failure of response inhibition when sustained attention is required for long periods of time (Watkins et al., 2000). P3A latencies, which may be used to examine the temporal components of attention (e.g., target salience, target detection; for review, Polich and Kok, 1995), were significantly delayed in HIV-1 seropositive adults; delays which increased in magnitude as a function of HIV-1 neurocognitive impairment (Fein et al., 1995). Alterations in sustained attention, assessed using the operant task of sustained attention employed in the present study, were also observed in ovariectomized, female HIV-1 Tg rats (Moran et al., 2014). HIV-1 Tg rats, relative to controls, required a significantly greater number of sessions to meet the criteria (i.e., 70% for three consecutive sessions) and displayed a decreased response rate, consistent with alterations in attention (Moran et al., 2014).

The contemporary phenotype of the non-infectious HIV-1 Tg rat, originally developed by Reid et al. (2001), contains a *gag-pol* deletion, has a mild neuroinflammatory environment, and resembles HIV-1 seropositive individuals on cART. Previous studies have established the utility of the HIV-1 Tg rat for examining neurocognitive deficits commonly observed in HAND, including deficits in executive function, sustained attention, temporal processing, spatial learning and episodic memory (e.g., Vigorito et al., 2007; Lashomb et al., 2009; Moran et al., 2013a,b, 2014; McLaurin et al., 2017a). To date, however, direct comparisons of neurocognitive deficits in female and male HIV-1 Tg rats have received little attention. Understanding the impact of biological sex on neurocognitive deficits in HIV-1 is crucial for the development of sex-based diagnostic screening tools, therapeutics and cure strategies.

## Materials and Methods

### Animals

Intact Fischer (F344/N; Harlan Laboratories Inc., Indianapolis, IN, USA) HIV-1 Tg (female, *n* = 11; male, *n* = 14) and control (female, *n* = 15; male, *n* = 16) animals were sampled from a total of 17 litters (HIV-1 Tg: *N* = 9; control: *N* = 8). Animals were delivered to the facility, housed with their biological dam, between postnatal day 7 and 9 over the course of 4 months. At postnatal day 21, animals were weaned, separated by sex, and pair- or group-housed. One week prior to beginning operant testing, animals were placed under food restriction (Pro-Lab Rat, Mouse, Hamster Chow #3000) to maintain them at 85% of their normal body weight. Water was available *ad libitum* throughout the duration of the study.

Training, including a shaping response protocol and a signal detection task, began at approximately 2 months of age. Given the failure to find an effect of estrous cycle stage on sustained attention performance (McGaughy and Sarter, 1999) and the protracted acquisition training (up to 113 days), estrous cycle stage was not examined. Performance on the signal detection task is unlikely to be sensitive to endogenous hormonal fluctuations occurring across the 4–4.5 days estrous cycle of the rat.

Animals were maintained in AAALAC-accredited facilities under the guidelines established by the National Institute of Health (NIH). Environmental conditions for the animal vivarium were targeted at 21° ± 2°C, 50% ± 10% relative humidity and a 12-h light: 12-h dark cycle with lights on at 07:00 h (EST). The study was carried out in accordance with the recommendations of the NIH. The project protocol was approved by the Institutional Animal Care and Use Committee of the University of South Carolina under federal assurance (#A3049-01).

### Apparatus

Behavioral training and assessments of sustained attention were conducted in 16 operant chambers, which were located inside sound-attenuating chambers (Med Associates, Inc., Fairfax, VT, USA). A pellet dispenser (45 mg) was located between two retractable levers on the front wall of the operant chambers. The front wall also included three panel lights, one located above each lever (not used in the present experiment) and one located above the pellet dispenser. A house light was located on the rear wall of the operant chamber. Signal presentation, lever operation, reinforcement delivery and data collection were controlled by a PC and Med-PC for Windows software (V 4.1.3; Med Associates, Inc., Fairfax, VT, USA).

### Shaping

A response shaping protocol, initiated at approximately 2 months of age, was used to train the animals to lever-press. Shaping was conducted using a 42-min test session. The house light was illuminated throughout the duration of the test session. A FR-1 schedule of reinforcement was used to train animals to press both levers, with sucrose pellets (45 mg) used for reinforcement. To prevent side bias, animals were limited to no more than five consecutive presses on a single lever. Successful acquisition of the task required animals to achieve at least 60 reinforcers for three consecutive or five non-consecutive days, at which point they were promoted to the signal detection task. Shaping continued until each and every animal was promoted, i.e., there was no censored data.

### Signal Detection

After successfully acquiring the shaping task, animals were trained in a stimulus detection task, including three vigilance programs, as described by McGaughy and Sarter (1995). All test sessions, conducted in a darkened operant chamber, began with a 5-min habituation period. Signal presentation (i.e., central panel light illumination vs. no illumination) was randomized across trials throughout the session. Levers were extended 2 s after each trial began and remained extended for 6 s for the animal to make a response. Levers were retracted between trials, which had intertrial intervals (ITI) of 9 ± 3 s. For half of the animals, lever presses on the left lever during signal trials and on the right lever during non-signal trials were rewarded with a sucrose pellet (hits and correct rejections, respectively). In the same manner, lever presses on the right lever during signal trials and on the left lever during non-signal trials were considered incorrect responses and were not rewarded with a sucrose pellet (misses and false alarms, respectively). The reverse set of rules was used for the other half of the animals. Animals were trained on each vigilance program until meeting criteria of at least 70% accuracy for five consecutive or seven non-consecutive test sessions, at which point they were promoted to the next program. Percent accuracy was calculated as follows: ((Number of Hits and Correct Rejections at 1000 ms)/(Number of False Alarms, Hits, Misses and Correct Rejections at 1000 ms)) × 100.

During the initial vigilance task, termination of the stimulus light was contingent upon a response. In the second vigilance program, the length of the stimulus light was 1 s. Both the first and second vigilance programs included 160 trials. During both vigilance programs, if an animal responded incorrectly, they were given correction trials, which included up to three repetitions of the trial. A forced-choice trial occurred if an animal failed to respond appropriately during the correction trials. During the forced-choice trial, the same stimulus (i.e., signal or non-signal) was presented, however, only the correct lever was extended. The correct lever remained extended for 120 s or until a correct response was made.

The third vigilance program manipulated the length of the stimulus. Stimulus length durations, which were block randomized across 162 trials, were 1000, 500, or 100 ms. Correction trials and force-choice trials were eliminated in the third vigilance task. Through all versions of signal detection, training continued until each and every animal met the criterion, i.e., there was no censored data.

### Statistical Analysis

Analyses were conducted using SAS 9.4 (SAS/STAT Software 9.4, SAS Institute, Inc., Cary, NC, USA). Due to the nested nature of the experimental design (i.e., rat pups within a litter), the litter was used as the unit of analysis for all statistical analyses (Denenberg, 1984). All figures display litter means, consistent with the statistical analysis. Figures displaying the acquisition of shaping and vigilance for biological sex are collapsed across genotype to directly examine the profound sex differences. An alpha level of *p* ≤ 0.05 was considered significant for all statistical tests.

The temporal process of task acquisition (i.e., the number of days to meet criterion) was assessed using two methods. First, a curve-fitting analysis, used to directly assess the functional form of temporal processes in task acquisition and fit with a 95% confidence interval (CI), was completed using GraphPad Prism 5 (GraphPad Software, Inc., La Jolla, CA, USA). Second, due to our interest in examining population level effects, a generalized estimating equation (GEE) model with a Poisson distribution was conducted. An unstructured covariance pattern was used. Between-subjects factors included in the analysis were genotype (HIV-1 Tg vs. Control) and biological sex (Male vs. Female).

Percent accuracy, calculated as ((Number of Hits and Correct Rejections at 1000 ms)/(Number of False Alarms, Hits, Misses, and Correct Rejections at 1000 ms)) × 100, was analyzed using a mixed-design analysis of variance (ANOVA), incorporating the evaluation of both between and within-subject factors. Additionally, a mixed-design ANOVA was used to analyze false alarms, hits, misses, and correct rejections. Genotype (HIV-1 Tg vs. Control) and biological sex (Male vs. Female) served as the between-subjects factor. Percent accuracy, day and response type (i.e., false alarms, hits, misses and correct rejections) served as the within-subjects factor, as appropriate. Independent samples *t*-tests were conducted to determine the locus of interactions observed in the mixed-design ANOVA examining response type. Effect sizes were calculated using partial eta squared ($\eta_{\text{p}}^{2}$), a measure of the variance accounted for with a maximum value of 1. Potential violations of compound symmetry were precluded by the use of orthogonal decomposition of the repeated-measures factors or addressed *post hoc* via the conservative Greenhouse-Geisser *df* correction factor (Greenhouse and Geisser, 1959).

## Results

### Shaping

#### Acquisition

**Figure 1:**
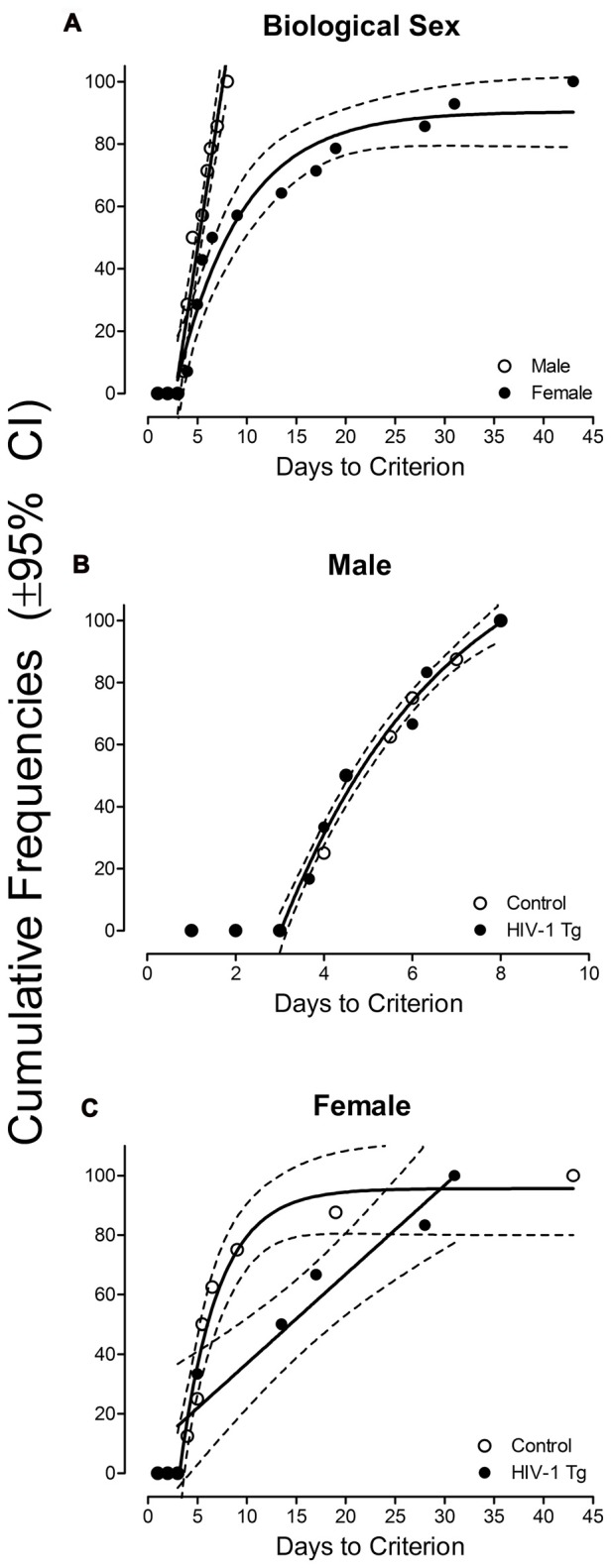
**(A)** The number of sessions required to meet criterion (at least 60 reinforcers for three consecutive or five non-consecutive days) in the shaping task is presented as a function of biological sex, representing both HIV-1 Tg and control animals by collapsing across genotype (±95% CI). A first-order polynomial provided a well-described fit for male animals (*R*^2^: 0.95), with all animals meeting criterion within 8 days. In sharp contrast, a one-phase association was the best fit for female animals (*R*^2^: 0.94). All female animals acquired the task within 43 days. **(B)** A global one-phase association was the best fit for male HIV-1 Tg and male control animals (*R*^2^: 0.98), suggesting no difference in the number of days to meet criterion between genotypes. **(C)** In female control animals, a one-phase association provided a well-described fit (*R*^2^: 0.96). However, in female HIV-1 Tg animals, shaping acquisition was best fit using a first-order polynomial (*R*^2^: 0.91). Presence of the HIV-1 transgene had a significant effect on the temporal acquisition of shaping in female, but not male, animals.

All HIV-1 Tg and control animals met the criteria of 60 reinforcers for three consecutive or five non-consecutive days. Biological sex had a significant effect on the number of days to acquire shaping, as illustrated in Figure 1A. All male animals, regardless of genotype, acquired the task within 8 days. In contrast, it took 43 days for all female animals to acquire the task. Curve-fitting analyses revealed that different curves best fit data from male and female animals. Specifically, a first-order polynomial was the best fit for male animals (*R*^2^: 0.95). In sharp contrast, a one-phase association provided a well-described fit for the number of days to acquire shaping in female animals (*R*^2^: 0.94). A GEE with a Poisson distribution confirmed our observations, revealing a significant main effect of sex (*z* = 2.22, *p* ≤ 0.027). On average, female animals acquired shaping 2.28 ± 1.82 (95% CI) times slower than male animals, holding all other variables constant. Thus, regardless of genotype, a greater number of sessions were required for female animals to acquire shaping.

Complementary results were obtained by conducting separate curve-fitting analyses of each sex, illustrated in Figure 1B (Male) and Figure 1C (Female). A global, one-phase association was the best fit for both male HIV-1 Tg and male control animals (*R*^2^: 0.98), indicating no significant genotype differences in the acquisition of shaping in male animals. In sharp contrast, the curve-fitting analysis suggested differences in the temporal process of acquisition in female animals dependent upon genotype. Shaping acquisition was best fit by a one-phase association for female control animals (*R*^2^: 0.96), while a first-order polynomial provided a well-described fit for female HIV-1 Tg animals (*R*^2^: 0.91). Specifically, 80% of the female control animals acquired shaping in approximately 10 sessions, while it took female HIV-1 Tg animals 25 sessions. Results provide compelling evidence for the effect of the HIV-1 transgene on the temporal acquisition of shaping in female animals, but not in male animals.

### Signal Detection

#### Acquisition

**Figure 2:**
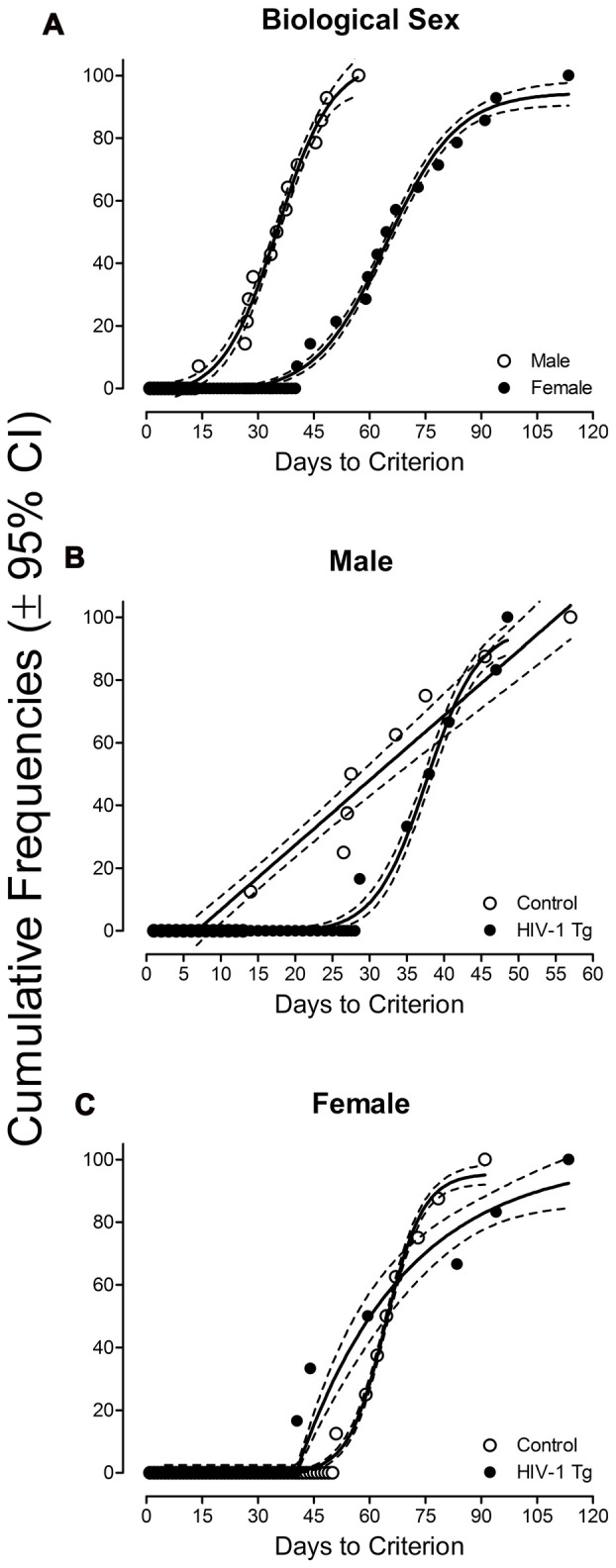
**(A)** The number of sessions required to meet criterion (70% accuracy for five consecutive or seven non-consecutive days) in the signal detection task is presented as a function of biological sex, representing both HIV-1 Tg and control animals by collapsing across genotype (±95% CI). A sigmoidal dose response was the best fit for both male (*R*^2^: 0.99) and female (*R*^2^: 0.99) animals, however, significant difference were observed in the fit of the function (*F*_(4,70)_ = 770.0, *p* ≤ 0.001). All male animals completed signal detection within 57 days. In stark contrast, female animals displayed a 27-day lag, relative to male animals, before beginning to acquire the task, with all animals meeting criterion within 113 days. **(B)** A first-order polynomial provided a well-described fit for male control animals (*R*^2^: 0.93), while a sigmoidal dose response curve was the best fit for male HIV-1 Tg (*R*^2^: 0.98). Male HIV-1 Tg animals exhibited a 14-day lag, relative to control animals, before beginning to acquire the signal detection task. **(C)** A sigmoidal dose response curve was the best fit for female control (*R*^2^: 0.99) animals. In sharp contrast, the number of test sessions required for female HIV-1 Tg animals to criterion were best fit with a plateau followed by a one-phase association (*R*^2^: 0.95). Overall, female control animals acquired the task at a significantly faster rate relative to female HIV-1 Tg animals.

All HIV-1 Tg and control animals were able to acquire the signal detection task, across a series of three programs, by meeting the criterion of 70% accuracy for five consecutive or seven non-consecutive days. In accordance with our observations in the acquisition of shaping, biological sex also had a significant effect on the number of days to acquire signal detection, illustrated in Figure 2A. A sigmoidal dose-response curve was the best fit for both male (*R*^2^: 0.99) and female (*R*^2^: 0.99) animals, however, significant differences in the fit of the function were observed (*F*_(4,70)_ = 770.0, *p* ≤ 0.001). Specifically, multiple parameters across the sigmoidal functions were significantly different dependent upon biological sex, including the top plateau (*F*_(1,70)_ = 5.8, *p* ≤ 0.02), the number of test sessions required for 50% of the animals to acquire the task (*F*_(1,70)_ = 67.6, *p* ≤ 0.001), and the steepness of the curve (*F*_(1,70)_ = 5.2, *p* ≤ 0.03). All male animals acquired the signal detection task within 57 days. In sharp contrast, female animals exhibited a 27-day lag, relative to male animals, before beginning to acquire the task. Overall, it took 113 days for all female animals to acquire the signal detection task, suggesting a profound sex difference in the acquisition of signal detection. Observations were confirmed using a GEE model with a Poisson distribution revealing a significant main effect of sex (*z* = 4.98, *p* ≤ 0.001). On average, female animals acquired the signal detection task 2.05 ± 0.58 (95% CI) times slower than male animals, holding all other variables constant.

Complementary results were obtained by conducting separate curve-fitting analyses of each sex, illustrated in Figure 2B (Male) and Figure 2C (Female). A first-order polynomial provided a well-described fit for male control animals (*R*^2^: 0.93), while a sigmoidal dose-response curve was the best fit for male HIV-1 Tg animals (*R*^2^: 0.98). Male HIV-1 Tg animals showed a 14-day lag, relative to male control animals, before beginning to acquire the task. For female control animals, a sigmoidal dose-response curve provided a well-described fit (*R*^2^: 0.99). In sharp contrast, the number of days to criterion for female HIV-1 Tg animals were best fit with a plateau followed by a one-phase association (*R*^2^: 0.95). The one-phase association began after 40.5 sessions (i.e., the mean number of sessions required for the first litter of HIV-1 Tg female animals to meet criterion). Although female control animals showed an initial 10-day lag in task acquisition, the overall rate of acquisition was significantly faster in comparison to female HIV-1 Tg animals. Specifically, 80% of the female control animals acquired the task in approximately 73 sessions, while it took female HIV-1 Tg animals 87 sessions.

#### Response Profile

**Figure 3:**
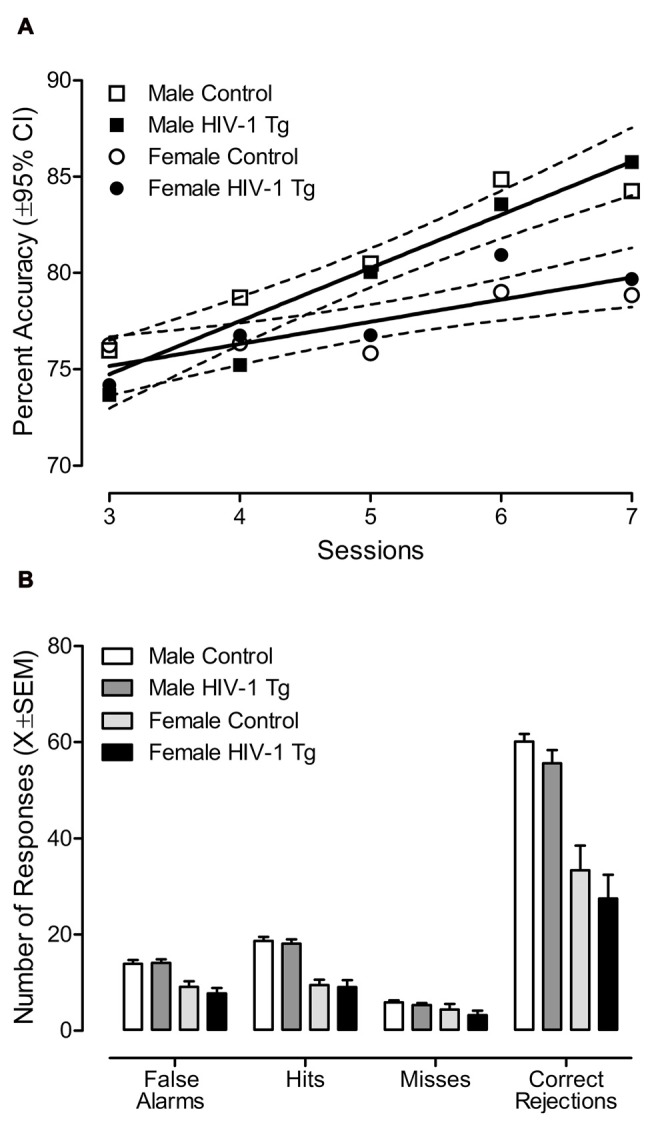
Once animal’s met criterion in the final signal detection task, the factor of biological sex had a significant effect on percent accuracy and an animal’s response profile. **(A)** Both male and female animals, regardless of genotype, exhibited a linear increase in percent accuracy across test sessions. However, the rate of increase in percent accuracy occurred more slowly in female animals relative to male animals. **(B)** Female animals displayed a decreased response rate relative to male animals on all response types (i.e., false alarms, hits, misses and correct rejections). There was no compelling evidence for any effect of the HIV-1 transgene on either percent accuracy or an animal’s response profile once the task was well acquired.

Once animals reached criterion in the final signal detection task, significant sex differences were observed in both percent accuracy (Figure 3A) and the response profile (Figure 3B).

Percent accuracy (Figure 3A) increased linearly in both male (*R*^2^: 0.90) and female (*R*^2^: 0.69) animals, independent of genotype, as a function of test session. Biological sex affected the rate of increase in percent accuracy across test sessions. Specifically, the rate of increase in percent accuracy occurred more slowly in female animals (*β*_1_ = 1.15 ± 0.63) relative to male animals (*β*_1_ = 2.76 ± 0.70). The mixed design ANOVA confirmed these observations, revealing a significant day × sex interaction (*F*_(4,96)_ = 3.11, *p_GG_* ≤ 0.032, $\eta_{\text{p}}^{2}$ = 0.115) with a prominent linear component (*F*_(1,24)_ = 6.9, *p* ≤ 0.015, $\eta_{\text{p}}^{2}$ = 0.224). Significant main effects of day (*F*_(4,96)_ = 18.1, *p_GG_* ≤ 0.001, $\eta_{\text{p}}^{2}$ = 0.430) and sex (*F*_(1,24)_ = 9.3, *p* ≤ 0.005, $\eta_{\text{p}}^{2}$ = 0.280) were also observed.

The factor of biological sex significantly affected the response profile (i.e., false alarms, hits, misses and correct rejections), illustrated in Figure 3B. A significant sex × response type interaction (*F*_(3,72)_ = 42.7, *p_GG_* ≤ 0.001, $\eta_{\text{p}}^{2}$ = 0.640) suggested that male and female animals exhibited significantly different response profiles. The overall ANOVA also revealed significant main effects of sex (*F*_(1,24)_ = 50.8, *p* ≤ 0.001, $\eta_{\text{p}}^{2}$ = 0.679) and response type (*F*_(3,72)_ = 406.7, *p_GG_* ≤ 0.001, $\eta_{\text{p}}^{2}$ = 0.944). Independent samples *t*-test were subsequently conducted to determine the locus of these interactions. Female animals exhibited fewer false alarms (*t*_(26)_ = −5.8, *p* ≤ 0.001), hits (*t*_(26)_ = −8.9, *p* ≤ 0.001), misses (*t*_(26)_ = −2.1, *p* ≤ 0.05) and correct rejections (*t*_(26)_ = −7.0, *p* ≤ 0.001) than male animals.

## Discussion

A signal detection operant task was conducted to examine the effect of biological sex on sustained attention deficits in the HIV-1 Tg rat. Robust sex differences were observed in the acquisition of shaping and signal detection. Female animals, regardless of genotype, acquired both the shaping and signal detection task significantly more slowly than male animals. In shaping, presence of the HIV-1 transgene had a significant effect on the temporal process of task acquisition in female, but not male, animals. In signal detection, presence of the HIV-1 transgene had a significant effect on task acquisition, with HIV-1 Tg animals acquiring the task significantly more slowly than control animals; a deficit which was more pronounced in female HIV-1 Tg animals. Once animals reached asymptotic performance in signal detection, the effect of biological sex was examined using percent accuracy and an animal’s response profile (i.e., false alarms, hits, misses and correct rejections). Female animals achieved a lower percent accuracy across test sessions and made significantly fewer responses compared to male animals. There was no compelling evidence for the effect of the HIV-1 transgene on percent accuracy and on an animal’s response profile once reaching asymptotic performance on the task. Results suggest that the factor of biological sex may be a moderator of the influence of the HIV-1 transgene on signal detection, which may be critical for the development of sex-based therapeutics and cure strategies.

Temporal processing deficits, which have been implicated as a potential elemental dimension of HAND, may underlie sustained attention deficits observed in the present study. Significant alterations in temporal processing, assessed using prepulse inhibition (PPI), have been well-defined and appear highly replicable in the HIV-1 Tg rat (e.g., Moran et al., 2013a; McLaurin et al., 2016, 2017a,b,d). Ovariectomized female HIV-1 Tg animals displayed alterations in the development of perceptual sharpening, assessed using auditory and visual PPI in a time-limited repeated measures assessment (Moran et al., 2013a). Studies in ovariectomized female HIV-1 Tg animals have also revealed the generality and relative permanence of temporal processing deficits in the HIV-1 Tg rat (McLaurin et al., 2017b,d). The effect of biological sex on the progression of temporal processing deficits was examined using PPI (McLaurin et al., 2017a) and gap PPI (McLaurin et al., 2016). HIV-1 Tg animals displayed profound alterations in the progression of temporal processing, deficits which were more pronounced in female HIV-1 Tg animals (McLaurin et al., 2016, 2017a). An assessment of the temporal processes inherent in sustained attention, such as stimulus duration, is fundamental to enhancing our understanding of temporal processing deficits in the HIV-1 Tg rat.

Neurocognitive deficits associated with HAND have been observed across the lifespan, from childhood (e.g., HIV-1 Tg rat: McLaurin et al., 2017c; HIV-1 seropositive individuals: Paramesparan et al., 2010; Cohen et al., 2015; Phillips et al., 2016) to adolescence (e.g., HIV-1 Tg rat: Moran et al., 2012; McLaurin et al., 2016; HIV-1 seropositive individuals: Willen et al., 2017) and older adulthood (i.e., >50 years; HIV-1 Tg rat: McLaurin et al., 2017a; HIV-1 seropositive individuals: Valcour et al., 2004; Fazeli et al., 2014; Sheppard et al., 2015). In the present study, HIV-1 Tg and control animals were assessed beginning at approximately 2 months of age, which is equivalent to young adulthood (i.e., approximately 18 years old) in humans (Sengupta, 2013); an age with strong translational relevance in the post-cART era. Following the advent of cART, HIV-1 transitioned from an acute, lethal disease to a chronic disease, which has enormous implications for neurocognitive development as HIV-1 seropositive children survive into adulthood (Sohn and Hazra, 2013; Crowell et al., 2014; Smith and Wilkins, 2015). Specifically, high rates of neurocognitive impairment, characterized by attentional and memory dysfunction, have been observed in children perinatally infected with HIV-1 surviving into adulthood (Paramesparan et al., 2010; Cohen et al., 2015; Phillips et al., 2016). By 2020, it is estimated that approximately 1.94 million children will be living with HIV-1 (Penazzato et al., 2014). Additionally, in the United States, approximately 22% of all new HIV-1 infections were in adolescents and young adults ranging in age from 13 to 24 years old (CDC, 2017), suggesting the importance of understanding neurocognitive deficits during this critical developmental period. Moreover, as the number of women diagnosed with AIDS continues to increase in the US and around the world, currently with almost 51% of infected adults being women, the magnitude of the pediatric population at risk will continue to challenge global resources (UNAIDS, 2017). Thus, examination of the HIV-1 Tg rat, which mimics the effect of long-term chronic exposure to HIV-1 viral proteins, beginning at 2 months of age (and continued until all animals acquired the task) provides an opportunity to understand neurocognitive deficits in adolescents and mature adults.

Evidence for the role of ovarian hormones, including estrogen, on selective neurocognitive tasks is complex and inconsistent (e.g., sustained attention: McGaughy and Sarter, 1999; spatial memory: Berry et al., 1997; Warren and Juraska, 1997; Frick and Berger-Sweeney, 2001; temporal processing: Koch, 1998; Adams et al., 2008). With specific reference to the sustained attention operant task, examination of estrous cycle revealed that performance did not vary across cycle stage (McGaughy and Sarter, 1999). Further, given the protracted acquisition training (up to 113 days), performance on the signal detection task is unlikely to be sensitive to endogenous hormonal fluctuations occurring across the 4–4.5 days estrous cycle of the rat. A meta-analysis further revealed that female animals are not more variable than male animals on neurocognitive behavioral assessments, even when estrous cycle stage is not accounted for Becker et al. (2016). Neurocognitive alterations may result from the effects of estrogen, which may act via multiple mechanisms, altering cerebral microvasculature, mitochondrial function, inflammation and cholinergic system function (review, Engler-Chiurazzi et al., 2017); mechanisms which may also contribute to the cognitive deficits in HAND.

To date, the precise neural mechanisms underlying the cognitive deficits in HAND is (are) unclear, however, multiple factors may be involved (Hong and Banks, 2015; Reid et al., 2016). Both clinical and preclinical studies have repeatedly reported evidence of neuroinflammation in HIV-1 (e.g., review: Appay and Sauce, 2008; HIV-1 seropositive humans: Meier et al., 2009; Royal et al., 2016; SCID mice: Sas et al., 2007; Boska et al., 2014; HIV-1 Tg rats: Royal et al., 2012; Rowson et al., 2016). Additionally, alterations in immunocompetence, which is influenced by estrogen (Fish, 2008), have been implicated as an underlying mechanism for sex differences in multiple neurodegenerative diseases (Hanamsagar and Bilbo, 2016). In HIV-1 seropositive individuals, sex differences have been observed in neuroinflammatory marker levels, including sCD14 and interferon α (INF-α; Meier et al., 2009; Royal et al., 2016). HIV-1 seropositive women exhibited increased, albeit not statistically significant, levels of sCD14 that were associated with neurocognitive impairment (Royal et al., 2016). Additionally, plasmacytoid dendritic cells from HIV-1 seropositive women, produced higher levels of INF-α following exposure to toll-like receptor ligands relative to HIV-1 seropositive men (Meier et al., 2009). Dopamine (DA) system dysfunction has also been implicated as a potential neural mechanism for neurocognitive impairment in HIV-1 (e.g., Wang et al., 2004; Chang et al., 2008; Webb et al., 2010; Kumar et al., 2011; Moran et al., 2012, 2013b, 2014; Lee et al., 2014; Reid et al., 2016). Most notably, increased neuronal injury in dopaminergic neurons may be driven by neuroinflammation (Gao et al., 2008; Tansey and Goldberg, 2010; Herrero et al., 2015). Thus, sex differences in neurocognitive impairment in the HIV-1 Tg rat may result from increased neuroinflammation, leading to increased neuronal injury in dopaminergic neurons.

One of the undeniable strengths of the present study was the lack of censored data, i.e., all animals successfully acquired the task, although that accomplishment took nearly 4 months. Type 1 censoring occurs when a subject is unable to acquire a task within a predetermined amount of time. In the present study, we decided *a priori* to train all animals in the signal detection task, regardless of the number of days required to meet criterion. If type 1 censoring had been employed after 60 days in the task, when all males had acquired the task, less than 40% of the female animals would have successfully acquired the signal detection task. Utilization of type 1 censoring would have precluded our ability to detect an effect of either biological sex or transgene, significantly limiting the inferences and generalizability of the present study. Although type 1 censoring may be necessary in some instances (e.g., longitudinal studies), the present study documents that, with persistence, both female and male animals are able to successfully complete the signal detection task.

The HIV-1 Tg rat, used in the present study, was originally introduced by Reid et al. (2001), containing seven of the nine HIV-1 genes. The contemporary derivation of the HIV-1 Tg rat is on an inbred F344/N background and has the transgene limited to chromosome 9. The non-infectious nature of the HIV-1 Tg rat makes it unsuitable for studies of infectivity or viral replication, however, it has been promoted for investigating the neurocognitive deficits commonly observed in HAND (e.g., Vigorito et al., 2007; Moran et al., 2013a; Repunte-Canonigo et al., 2014; Reid et al., 2016; McLaurin et al., 2017a). HIV-1 Tg rats in the present study displayed no significant health disparities, consistent with our previous studies (Moran et al., 2012, 2013b; Roscoe et al., 2014; McLaurin et al., 2017a). Strong evidence for the integrity of visual system function through the majority of the animal’s functional lifespan has been reported in visual PPI, suggesting that animals are capable of detecting brightness (Moran et al., 2013a; McLaurin et al., 2017a). Additionally, both HIV-1 Tg and control animals display significant ambulatory activity, assessed using locomotor activity, providing no compelling evidence for gross-motoric system impairments through the majority of an animal’s functional lifespan (Moran et al., 2013b; McLaurin et al., 2017a) and a clear absence of any hind-limb paralysis. Therefore, the contemporary phenotype of the HIV-1 Tg rat closely resembles HIV-1 seropositive individuals on cART and is a valuable tool for examining sustained attention deficits in HIV-1.

In conclusion, the factor of biological sex significantly influenced acquisition and the response profile in a signal detection task used to tap sustained attention deficits. Female animals, regardless of genotype, took significantly more sessions to acquire the task and had a decreased response rate relative to male animals. As has been well-established for many nonreproductive behaviors, sex differences were anticipated to be small in magnitude (Goy and McEwen, 1980), therefore, the robust size of the sex difference in sustained attention performance was unexpected. Genotype (i.e., HIV-1 Tg vs. control) affected the number of sessions required to acquire the signal detection task, but had no significant effect on an animals’ response profile once the animals’ reached asymptotic performance. Results suggest that the factor of biological sex may be a moderator of the influence of the HIV-1 transgene on signal detection. Addressing sex differences in HAND does not distract from, but actually enriches, the primary scientific interest of developing cure strategies for HIV-1.

## Author Contributions

RMB and CFM: conceived and designed the experiments. KAM, RMB and CFM: performed the experiments. KAM, AJF and CFM: analyzed the data. KAM, CFM and RMB: wrote the article. KAM, RMB, CFM and AJF: critical appraisal and approval of final manuscript.

## Conflict of Interest Statement

The authors declare that the research was conducted in the absence of any commercial or financial relationships that could be construed as a potential conflict of interest.
